# Diabetic Hyperglycemia Induces Region-Specific Transcriptomic Remodeling in the Cochlea

**DOI:** 10.3390/genes17070836

**Published:** 2026-07-21

**Authors:** Ting-Yu Chang, Cheng-Tien Wu, Fong-Ling Chung, Shing-Hwa Liu, Ting-Hua Yang

**Affiliations:** 1 Institute of Toxicology, College of Medicine, National Taiwan University, Taipei 10051, Taiwan; d03447003@ntu.edu.tw; 2 Department of Nutrition, China Medical University, Taichung 406040, Taiwan; ct-wu@mail.cmu.edu.tw; 3 Department of Otolaryngology, College of Medicine and Hospital, National Taiwan University, Taipei 10051, Taiwan; iwishlin@gmail.com; 4 Department of Medical Research, China Medical University Hospital, China Medical University, Taichung 406040, Taiwan; 5 Department of Pediatrics, College of Medicine and Hospital, National Taiwan University, Taipei 10051, Taiwan

**Keywords:** diabetes mellitus, cochlea, ototoxicity, RNA sequencing, hearing loss

## Abstract

Background/Objectives: Diabetes mellitus is associated with sensorineural hearing loss, but the cochlear molecular alterations related to this complication remain unclear. This pilot study aimed to characterize auditory function and region-specific transcriptomic changes in the diabetic cochlea. Methods: A streptozotocin-induced type 1 diabetic mouse model was established. Auditory function was assessed by auditory brainstem response (ABR) testing. Next-generation RNA sequencing was performed on micro-dissected cochlear regions, including the modiolus, lateral wall, and organ of Corti, followed by differential expression and gene ontology analyses. Results: STZ-induced diabetic mice showed elevated ABR thresholds compared with control mice. RNA sequencing revealed region-specific transcriptomic alterations across cochlear regions, with the lateral wall showing the greatest changes. Cross-region analysis identified a shared transcriptional signature consisting of 32 upregulated and 2 downregulated genes across the modiolus, lateral wall, and organ of Corti. Gene Ontology analysis showed enrichment of immune-related, inflammatory, metabolic stress-associated, and membrane-associated signaling. Conclusions: STZ-induced diabetes was associated with ABR threshold elevation and shared as well as region-specific cochlear transcriptomic alterations in this mouse model. These findings provide a region-specific cochlear transcriptomic resource for diabetes-associated auditory dysfunction and support future studies using larger cohorts, independent molecular validation, histological assessment, and cell-type-resolved approaches.

## 1. Introduction

The global epidemic of diabetes mellitus (DM) has become one of the most critical public health challenges of the 21st century. With the advancement of modern technology and changes in lifestyle, the number of individuals diagnosed with diabetes is rising. According to the World Health Organization (WHO), the global diabetic population increased from 200 million in 1990 to 830 million in 2022 [1]. This upward trend is evident not only in high-income countries such as Japan, the United States, and those in Europe but is also accelerating rapidly in low- and middle-income nations [2]. According to WHO (2024), in 2017, approximately 9 million people suffered from type 1 diabetes (insulin-dependent) [1]. More than 95% of diabetic patients have type 2 diabetes (non-insulin-dependent).

The high prevalence of diabetes imposes a substantial burden on global healthcare systems and economies. The global expenditure for the treatment of diabetes and its related complications has reached approximately USD 727 billion, accounting for nearly 12% of total global healthcare costs [3,4]. In Taiwan, data from the Ministry of Health and Welfare indicate that diabetes remains a major chronic disease, ranking fifth in mortality (as of 2018), with a prevalence of 8–10%, and ranking second in National Health Insurance outpatient expenditures. Diabetes can result in a range of severe complications, including cerebrovascular accidents, stroke, cardiovascular disease, nephrotic syndrome, diabetic ketoacidosis, peripheral neuropathy, and hearing loss [5,6]. Type 1 diabetes is primarily caused by genetic predisposition or autoimmune destruction of pancreatic β-cells, leading to absolute insulin deficiency. Patients require lifelong and regular insulin therapy [5]. The absence of insulin results in elevated blood glucose levels and accumulation of advanced glycation end-products (AGEs), which disrupt carbohydrate, protein, and lipid metabolism. Long-term glycemic dysregulation can lead to acute ketoacidosis, nephropathy, peripheral vascular disease, and neuropathy. While insulin therapy remains the cornerstone of treatment for Type 1 diabetes, environmental factors such as viral infections, dairy consumption, inadequate sunlight exposure, oxidative stress, chronic inflammation, and exposure to chemicals like pentamidine may accelerate the onset and severity of diabetic complications [5].

Numerous studies have demonstrated a strong correlation between the progression of diabetes and hearing impairment [7,8,9]. Hyperglycemia-induced microvascular damage is a key pathological feature of diabetes, affecting the retina, cardiovascular system, kidneys, and peripheral nerves. Similarly, the cochlea may be affected by compromised microvasculature, leading to insufficient oxygen and nutrient supply, which in turn damages hair cells and auditory nerve fibers, ultimately contributing to sensorineural hearing loss [10,11,12]. Additionally, diabetes-induced peripheral neuropathy may impair auditory nerve fibers. Hyperglycemia and calcium dysregulation may further injure cochlear neurons, impairing signal transduction and auditory processing [13]. Research suggests that hearing impairment in diabetes may result from the combined effects of microangiopathy and neuropathy. Assessments such as distortion product otoacoustic emissions (DPOAE) and auditory brainstem response (ABR) testing have demonstrated that diabetes-related hearing loss is associated with cochlear microvascular dysfunction and impaired neural conduction [14].

Despite increasing evidence of this association, there remains a lack of systematic investigation into the independent or synergistic mechanisms underlying diabetes-induced hearing loss. Therefore, the present study was designed as a pilot study in a diabetic mouse model and aimed to utilize next-generation sequencing (NGS) technology to comprehensively investigate the molecular mechanisms through which diabetes contributes to auditory dysfunction. By elucidating these pathways, this research seeks to provide valuable scientific insights to support future clinical strategies for the prevention and treatment of diabetes-related hearing loss.

## 2. Materials and Methods

### 2.1. Establishment of Streptozotocin (STZ)-Induced Type 1 Diabetic Mouse Model

Four-week-old male C57BL/6J mice (20–25 g) were obtained from the Laboratory Animal Center of National Taiwan University College of Medicine. Animals were housed under controlled environmental conditions (temperature: 22 ± 1.5 °C; humidity: 40–60%) with a 12 h light/dark cycle. To induce Type 1 diabetes, mice in the experimental group were intraperitoneally injected with freshly prepared streptozotocin (STZ; MilliporeSigma, Burlington, MA, USA; 50 mg/kg in 0.01 M citrate buffer, pH 4.5) for five consecutive days. Control mice received equivalent volumes of 0.01 M citrate buffer (*n* = 8 per group). One week after the final injection, mice with blood glucose levels ≥ 250 mg/dL were considered diabetic and selected for further study [15]. Blood glucose levels were measured again at the time of sacrifice to confirm persistent hyperglycemia. Hearing function was assessed at week 20 post-induction. Diabetic mice showing significant auditory threshold shifts compared to controls were included for further analyses.

An animal study was performed following the guidelines of the Association for Assessment and Accreditation of Laboratory Animal Care International (AAALAC) and the NIH (National Research Council) Guide for the Care and Use of Laboratory Animals. The animal experiments conformed to animal protocol and procedures approved by the Institutional Animal Care and Use Committee at the National Taiwan University College of Medicine (IACUC approval NO.: 20220320).

### 2.2. Auditory Brainstem Response Testing (ABR)

Auditory thresholds were assessed using standard ABR recording procedures. Mice were anesthetized by intraperitoneal injection of pentobarbital (35 mg/kg) and placed on a thermostatically controlled heating pad to maintain body temperature. Subdermal stainless-steel needle electrodes were positioned at the vertex (active), behind the test ear (reference), and at the neck (ground). Acoustic stimuli, including broadband clicks and tone bursts at 4, 8, 16, and 32 kHz, were delivered through a calibrated plastic acoustic tube coupled to the test ear (SmartEP2 system, Intelligent Hearing Systems, Miami, FL, USA). Tone bursts were presented with a 1 ms rise/fall time and a 3 ms plateau. For each stimulus condition, ABR waveforms were generated from the averaged responses of 1000 sweeps. Thresholds were determined by reducing stimulus intensity in 5–10 dB steps and identifying the lowest level at which a repeatable wave I or overall ABR waveform could be reliably detected.

### 2.3. RNA Isolation and Library Construction

After auditory testing, mice were sacrificed, and fresh cochlear tissues were harvested and dissected under a stereomicroscope into three compartments: lateral wall (including spiral ligament and stria vascularis), organ of Corti, and modiolus. Total RNA was extracted from each sample using the RNeasy Mini Kit (Qiagen, Singapore). RNA quality and concentration were evaluated using the Agilent 2100 Bioanalyzer (Agilent Technologies, Santa Clara, CA, USA), NanoDrop spectrophotometry (Thermo Fisher, Waltham, MA, USA), and 1% RNA gel electrophoresis. Only samples with RNA integrity number (RIN) > 7 were used. A total of 2 μg RNA per sample was subjected to sequencing library preparation using the Illumina^®^ NEBNext^®^ Ultra™ RNA-seq library kit; ribosomal RNA was removed using Ribo-Zero™ (Illumina, San Diego, CA, USA), followed by cDNA synthesis with ProtoScript II reverse transcriptase and second-strand synthesis using dACGTP/dUTP. Purified double-stranded cDNA was end-repaired, A-tailed, and ligated with indexed adapters. After size selection (insert size ~300 bp), the libraries were amplified, multiplexed, and sequenced on an Illumina HiSeq platform (2 × 150 paired-end). Sequencing data were processed by GENEWIZ. For each cochlear region, RNA-seq was performed using three biological replicates from control mice and three biological replicates from STZ-induced diabetic mice.

### 2.4. Data Filtering, Quality Control and Differential Gene Expression Analysis in Next-Generation Sequencing

Raw FASTQ reads were trimmed using Trimmomatic v0.30 to remove adapter sequences, low-quality bases, and PCR artifacts. High-quality reads were retained for downstream analysis. Reference genomes and annotations were obtained from UCSC, NCBI, and ENSEMBL databases. HISAT2 v2.0.1 was used to index and align reads to the reference genome. Gene expression was quantified using HTSeq v0.6.1. Differential expression analysis of mRNAs was conducted using the DESeq Bioconductor package, based on a negative binomial model. Genes with a fold change > 4 (|log_2_FC| > 2) and an adjusted *p*-value < 0.05 (Benjamini–Hochberg correction) were considered significantly differentially expressed. Potential batch effects were evaluated by examining sample clustering using principal component analysis. RNA extraction, library preparation, sequencing, and primary data processing were performed using the same workflow across samples, and no known technical batch factor was identified. Because PCA did not reveal clustering attributable to an apparent technical batch, no additional batch-correction procedure was applied prior to differential expression analysis. Gene Ontology (GO) and Kyoto Encyclopedia of Genes and Genomes (KEGG) pathway analyses were conducted using GO-TermFinder (an open source software; http://search.cpan.org/dist/GO-TermFinder/, accessed on 11 June 2026) [16]. Multiple-testing correction was performed using Bonferroni correction, and GO terms with corrected *p* < 0.05 were considered significantly enriched. The raw data files of the RNA-seq data have been uploaded to the Gene Expression Omnibus (GEO) repository (GSE303895).

### 2.5. Statistical Analysis

All experiments were performed in at least three independent replicates. Data are presented as mean ± standard deviation (SD). Statistical analyses were conducted using GraphPad Prism version 8 (GraphPad Software, San Diego, CA, USA). Comparisons between groups were performed using the Mann–Whitney U test (non-parametric) or one-way analysis of variance (ANOVA), as appropriate. A *p*-value < 0.05 was considered statistically significant.

## 3. Results

### 3.1. STZ-Induced Diabetic Mice Exhibit Metabolic Abnormalities and Auditory Dysfunction

**Figure 1:**
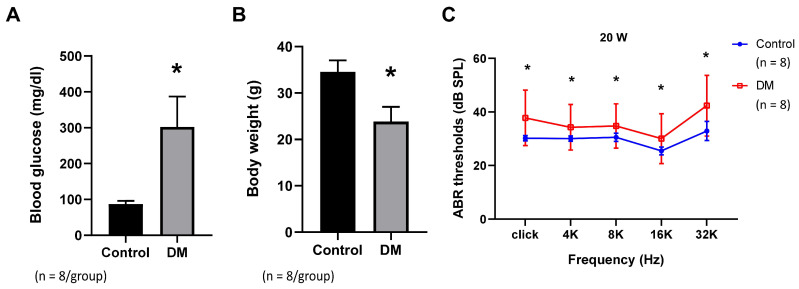
STZ-induced diabetic mice exhibit metabolic abnormalities and auditory dysfunction. (**A**) Blood glucose levels and (**B**) body weight measured at the time of sacrifice in control and STZ-induced diabetic (DM) mice. (**C**) ABR thresholds measured using click stimuli and tone bursts at 4, 8, 16, and 32 kHz at 20 weeks after STZ induction. Data are shown as mean ± SD; *n* = 8 mice per group. Statistical analysis was performed using two-way ANOVA. A significant group effect was observed between control and diabetic mice (*, *p* < 0.0001), with a significant group × stimulus condition interaction (*p* = 0.0013).

To establish a type 1 diabetic mouse model, C57BL/6J mice were intraperitoneally injected with streptozotocin (STZ). One week after the final STZ injection, mice with blood glucose levels ≥ 250 mg/dL were considered diabetic and selected for further study. At the experimental endpoint, STZ-induced diabetic mice showed markedly elevated blood glucose levels compared with control mice (87.33 ± 8.64 vs. 302.1 ± 8499 mg/dL, *p* < 0.0001; Figure 1A), confirming sustained hyperglycemia in the diabetic group. Compared with control mice, STZ-treated mice showed significantly reduced body weight (Figure 1B, *p* < 0.05). These physiological alterations are consistent with the catabolic phenotype associated with insulin deficiency and confirm the successful establishment of the diabetic model. To evaluate whether diabetes affected auditory function, auditory brainstem response (ABR) thresholds were measured using click stimuli and tone bursts at 4, 8, 16, and 32 kHz at 20 weeks after STZ induction. A total of eight mice per group were included in the ABR analysis (Figure 1C). Two-way ANOVA revealed a significant overall group effect between control and diabetic mice (F(1, 870) = 144.0, *p* < 0.0001), with diabetic mice showing a higher least-squares mean ABR threshold than control mice. The analysis also showed a significant stimulus condition effect (F(4, 870) = 40.16, *p* < 0.0001) and a significant group × stimulus condition interaction (F(4, 870) = 4.523, *p* = 0.0013), indicating that diabetes-associated threshold elevation differed across the tested stimulus conditions. These findings support the presence of auditory dysfunction in STZ-induced diabetic mice.

### 3.2. Diabetes Induces Region-Specific Transcriptional Divergence Across Cochlear Compartments

**Figure 2:**
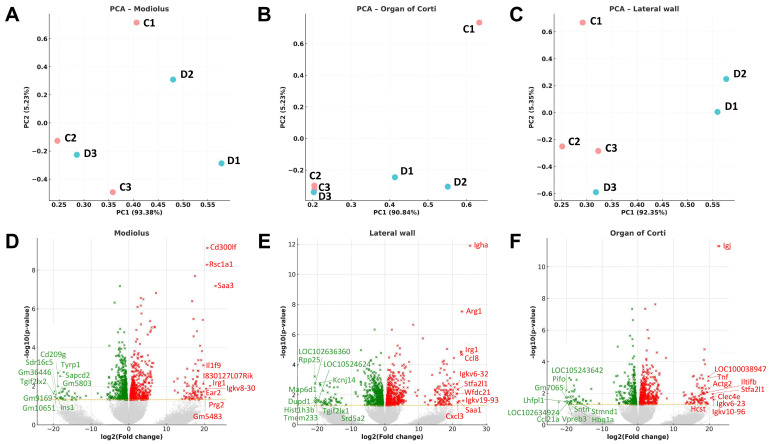
PCA and differential gene expression in cochlear regions. (**A**–**C**) Principal component analysis (PCA) of RNA-seq profiles from the modiolus (**A**), lateral wall (**B**), and organ of Corti (**C**). The *x*- and *y*-axes indicate PC1 and PC2, respectively, with the percentage of total variance explained by each principal component shown in parentheses. Control (C1–C3) and diabetic (D1–D3) samples segregate distinctly across the first two principal components. (**D**–**F**) Volcano plots showing differential gene expression in the modiolus, lateral wall, and organ of Corti. The *x*-axis represents log_2_ fold change, and the *y*-axis represents −log_10_(*p*-value). Significantly upregulated and downregulated genes were defined using adjusted *p* < 0.05 and |log_2_ fold change| > 2. The dashed yellow line is the threshold for statistical significance. Red dots indicate significantly upregulated genes, green dots indicate significantly downregulated genes, and gray dots indicate genes that did not meet the DEG criteria.

To characterize the molecular effects of diabetes on the auditory system, RNA sequencing was performed on three micro-dissected cochlear regions, including the modiolus, lateral wall, and organ of Corti, from diabetic and control mice. Principal component analysis (PCA) was used to visualize global transcriptomic variation among samples. The percentages of total variance explained by PC1 and PC2 are indicated on the corresponding axes in Figure 2A–C. It showed clear separation between diabetic and control samples in all three cochlear regions (Figure 2A–C), indicating diabetes-induced transcriptional remodeling across cochlear compartments. Among these regions, the lateral wall showed the most pronounced and consistent separation, with diabetic samples clustering distinctly from controls along PC1. This finding suggests that the lateral wall may be particularly sensitive to diabetes-associated metabolic and inflammatory stress. The modiolus and organ of Corti also displayed diabetic–control separation, although with greater inter-individual variability, suggesting region-dependent differences in transcriptional responses to hyperglycemia.

Volcano plot analyses further revealed widespread differential gene expression across the three cochlear regions (Figure 2D–F). The lateral wall exhibited the greatest number and magnitude of differentially expressed genes (DEGs), characterized by marked upregulation of immune- and inflammation-related genes, including *Arg1*, *Ccl8*, and *Saa3*, and downregulation of genes associated with membrane transport or structural regulation, including *Klk6*, *Ttc9b*, and *Cacng2*. In the modiolus, diabetes induced prominent upregulation of genes involved in immune activation and metabolic stress, including *Saa3*, *Irg1*, and *Cd300lf*, together with downregulation of transcripts related to neuronal signaling and synaptic support. In the organ of Corti, diabetes resulted in moderate but significant transcriptional changes, including upregulation of *Ccl8*, *Cd300lf*, and *Irg1*, and downregulation of genes associated with cytoskeletal organization and sensory epithelial maintenance, such as *Klk6*, *Olig1*, and *Cdk5r2*.

### 3.3. Top-Ranked Differentially Expressed Genes Reveal Region-Specific Molecular Signatures in the Diabetic Cochlea

**Figure 3:**
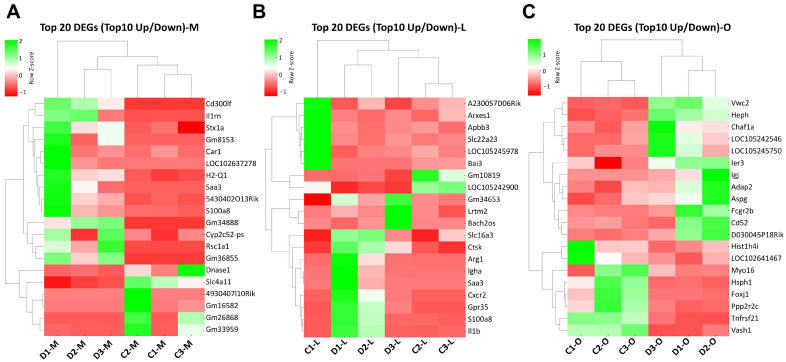
Top 10 differentially expressed genes (Top10 Up/Top10 Down) across cochlear regions. (**A**–**C**) Clustered heatmaps of the top 10 upregulated and top 10 downregulated genes (ranked by |log_2_FC|) in the modiolus (**A**), lateral wall (**B**), and organ of Corti (**C**). Expression values represent pseudo-count-corrected FPKM transformed into row-wise Z-scores. Bright red indicates relative upregulation; bright green indicates downregulation. Hierarchical clustering reveals clear group separation between control and diabetic samples, highlighting region-specific transcriptional alterations.

To identify the most prominent molecular features associated with diabetes-induced cochlear pathology, the top 10 upregulated and top 10 downregulated genes in each cochlear region were visualized by hierarchical clustering (Figure 3A–C). The resulting heatmaps showed clear segregation between diabetic and control samples across all three regions, further supporting substantial diabetes-induced transcriptomic remodeling.

Among the examined regions, the lateral wall displayed the most distinct clustering pattern, with differentially expressed genes primarily associated with inflammation, metabolic dysregulation, and membrane remodeling. In the modiolus, the DEG signature reflected enrichment of immune-related transcriptional programs and neuronal stress responses, whereas the organ of Corti showed transcriptional alterations related to sensory epithelial function, cytoskeletal organization, and cellular stress. These findings indicate that diabetes induces compartment-specific transcriptional signatures and highlight distinct regional vulnerabilities within the diabetic cochlea.

### 3.4. Gene Ontology Analysis Reveals Shared and Region-Specific Pathways Altered by Diabetes

**Figure 4:**
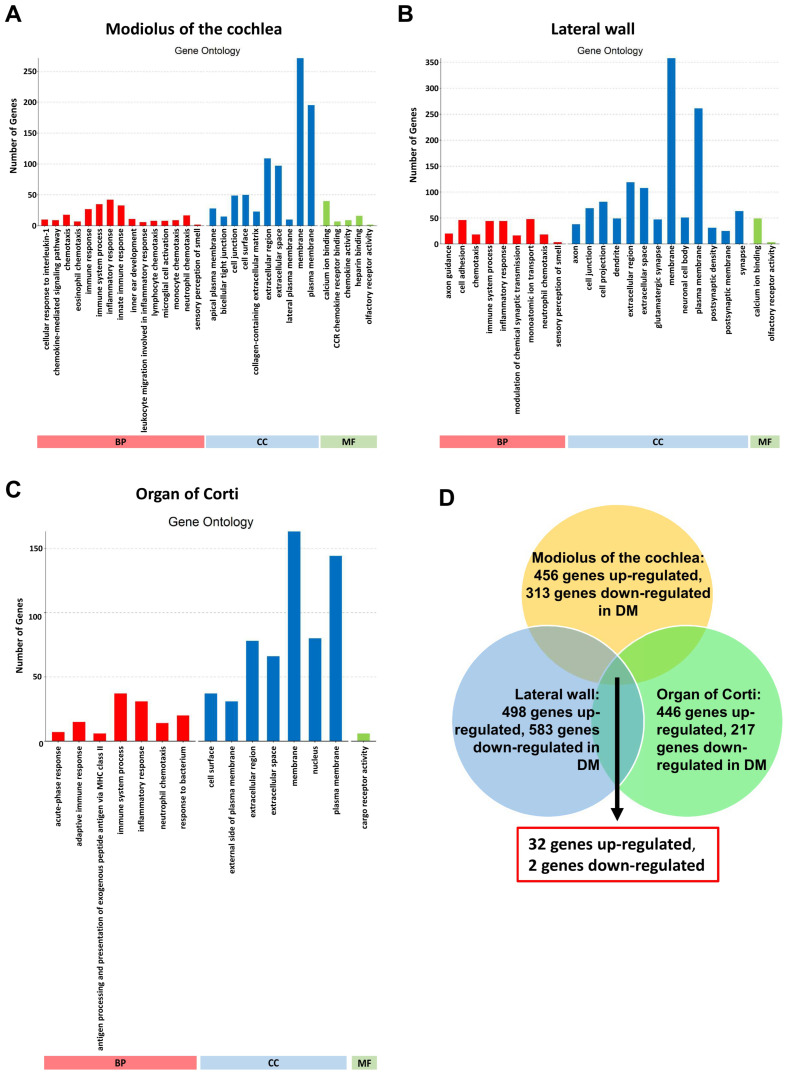
Functional enrichment and shared DEG signatures across cochlear regions. (**A**–**C**) Gene Ontology (GO) analysis of DEGs from the modiolus, lateral wall, and organ of Corti, categorized into biological processes (BP), cellular components (CC), and molecular functions (MF). (**D**) Venn diagram summarizing DEG overlap across regions. A total of 32 genes were consistently upregulated and 2 genes consistently downregulated across all cochlear regions in diabetic mice, representing a shared molecular signature of diabetes-induced cochlear dysfunction.

Gene Ontology (GO) enrichment analysis was performed to determine the biological processes, cellular components, and molecular functions associated with diabetes-induced DEGs in each cochlear region. Across the modiolus, lateral wall, and organ of Corti, DEGs were prominently enriched in immune-related biological processes, including inflammatory response, chemotaxis, and cytokine-mediated signaling pathways (Figure 4A–C). Enriched cellular component terms were primarily associated with membrane-associated and extracellular structures, suggesting remodeling of intercellular communication under hyperglycemic stress. Molecular function categories included calcium ion binding and receptor activity, consistent with perturbations in ion regulation and cell signaling.

Quantitative analysis of DEG distribution demonstrated that the lateral wall exhibited the greatest transcriptomic burden, with 498 upregulated and 583 downregulated genes, followed by the modiolus and organ of Corti. Cross-region comparison identified 32 genes that were consistently upregulated and 2 genes that were consistently downregulated across all three cochlear regions (Figure 4D and Table 1). The shared upregulated genes, including *Saa3*, *Irg1*, *Il1rn*, *Cd300lf*, *Stfa2l1*, and *Igj*, represent a conserved inflammatory and metabolic stress-related transcriptional response. In contrast, the two commonly downregulated genes, *Hspa1b* and *Lrrc73*, may reflect impaired cellular stress adaptation and altered membrane-associated structural or regulatory functions. Together, this shared DEG signature suggests the presence of a conserved hyperglycemia-responsive transcriptional program across the diabetic cochlea.

Collectively, these findings demonstrate that diabetes induces robust transcriptomic remodeling throughout the cochlea, characterized by inflammation, metabolic stress, and disruption of membrane and structural homeostasis. The lateral wall appears to be the most transcriptionally responsive region, whereas the modiolus and organ of Corti exhibit substantial but more heterogeneous transcriptional changes. These shared and region-specific molecular signatures provide mechanistic insight into diabetes-associated cochlear dysfunction and may help identify candidate biomarkers or therapeutic targets for diabetes-related hearing loss.

## 4. Discussion

Hearing loss is a well-documented but underappreciated complication of diabetes mellitus, with numerous clinical and experimental studies reporting progressive sensorineural hearing deficits in diabetic individuals. However, despite growing recognition, the molecular mechanisms underlying diabetes-induced auditory dysfunction remain largely unresolved. Our study provides a comprehensive analysis combining functional and transcriptomic evidence to elucidate the cochlear alterations associated with type 1 diabetes, and frames these findings within the context of auditory toxicology.

Using an STZ-induced type 1 diabetic mouse model, we observed elevated ABR thresholds in diabetic mice compared with controls. Two-way ANOVA showed a significant overall group effect, indicating higher ABR thresholds in diabetic mice across the tested stimulus conditions. A significant group × stimulus condition interaction further suggested that the effect of diabetes on ABR thresholds varied across click and tone-burst frequencies. These functional changes are consistent with previous reports showing hearing loss in both human patients with diabetes and diabetic rodents [17,18,19]. However, because only ABR testing was performed, the present data do not distinguish among sensory hair cell dysfunction, neural conduction deficits, or other cochlear functional changes. Additional measurements, such as distortion product otoacoustic emissions, cochlear action potentials, histological analysis, hair cell counts, spiral ganglion neuron counts, and stria vascularis morphology, will be required to define the cellular and structural basis of the observed auditory phenotype.

A major finding of this study is the prominent enrichment of inflammation- and immune-related transcriptional signatures across cochlear regions in diabetic mice. Rather than implicating a single gene or cell type, the RNA-seq data suggest a coordinated transcriptional response involving inflammatory signaling, chemokine-related pathways, immune-associated transcripts, and metabolic stress-related genes. This approach revealed extensive and region-specific transcriptomic reprogramming in diabetic cochleae, highlighting both shared and unique gene expression signatures that were previously unexplored. The lateral wall exhibited the highest number of differentially expressed genes (DEGs), suggesting that this region may be particularly responsive to sustained hyperglycemia in the STZ model. This aligns with prior hypotheses proposing the lateral wall as an early target in diabetic ototoxicity [19].

Among the upregulated genes consistently detected across all cochlear regions in diabetic mice are *Saa3*, *Irg1*, *Gm10872*, *Il1rn*, *Stfa2l1*, and *Igj*, many of which are implicated in inflammatory or immune responses. *Saa3*, a pro-inflammatory acute-phase protein primarily studied in metabolic and pulmonary inflammation, was prominently induced [20]. Although *Saa3* has not been previously reported in the inner ear, its strong expression suggests a potential role in initiating or amplifying cochlear inflammatory responses under hyperglycemic stress. Similarly, *Irg1* (*Acod1*), known for its role in itaconate production and immunometabolic regulation in macrophages, was upregulated in diabetic cochlear tissue [21]. This finding implies a previously unrecognized metabolic-inflammatory crosstalk in the cochlea, possibly involving immune cell activation or oxidative stress modulation. Anti-inflammatory mediators were also induced. Notably, both *Il1rn* and *Il1r2*, encoding the interleukin-1 receptor antagonist and decoy receptor, respectively, were upregulated, suggesting a compensatory attempt to suppress excessive IL-1 signaling and maintain cochlear homeostasis [22]. In addition, several poorly characterized or unannotated genes emerged as robustly upregulated. *Gm10872*, a predicted transcript with no known functional annotation, may represent a novel diabetes-responsive gene in cochlear tissue. *Stfa2l1*, a cystatin-like protease inhibitor, may be involved in modulating proteolytic activity or immune processing [23], although its role in the auditory system remains undefined. *Igj*, encoding the immunoglobulin J chain, is essential for polymeric IgA and IgM secretion [24], and its induction could signify increased local humoral immune activation or B-cell infiltration in the cochlea, which is an unusual but noteworthy finding with possible relevance to autoimmune or inflammatory inner ear disease. In contrast to the upregulated pro-inflammatory and immune response genes, only two genes were consistently downregulated across all cochlear regions: *Hspa1b* and *Lrrc73*. *Hspa1b* encodes a member of the Hsp70 family of heat shock proteins, which play crucial roles in protein folding, cellular protection from oxidative stress, and cytoprotection against apoptotic stimuli [25]. Downregulation of *Hspa1b* in diabetic cochlear tissue may suggest an impaired cellular stress response, potentially exacerbating cochlear vulnerability to hyperglycemia-induced oxidative damage. A previous study has shown that Hsp70 family members are upregulated in response to acoustic trauma and aminoglycoside-induced ototoxicity, where they exert protective effects on sensory hair cells and cochlear neurons [26]. Thus, the reduction of *Hspa1b* in diabetic cochleae may contribute to the progression of hearing loss by limiting intrinsic cytoprotective mechanisms. *Lrrc73* (Leucine-rich repeat-containing 73), though poorly characterized, belongs to a family of structural or scaffold proteins. Its downregulation may reflect disruption of epithelial cell architecture or altered cellular signaling within the cochlear lateral wall or organ of Corti. Given the importance of cytoskeletal integrity in maintaining hair cell polarity and stereocilia function, *Lrrc73* suppression may also play a role in the structural deterioration associated with diabetic cochlear injury, although further investigation is required to elucidate its specific function. Together, the downregulation of *Hspa1b* and *Lrrc73* may reflect a broader attenuation of cellular stress adaptation and cytoskeletal maintenance mechanisms in the diabetic cochlea, possibly exacerbating the inflammatory and metabolic injury pathways identified in this study. Collectively, these findings not only confirm the activation of canonical inflammatory and immune pathways in diabetic cochlear injury, but also highlight previously unrecognized transcripts that may serve as novel molecular markers or candidate pathways for future mechanistic validation in metabolic ototoxicity.

Importantly, many of the DEGs identified here have not been previously reported in the context of diabetes-related auditory dysfunction, highlighting the novelty of our transcriptomic approach. Moreover, the overlap of injury signatures with those described in drug- and noise-induced ototoxicity provides compelling evidence that chronic hyperglycemia can mimic toxicant-induced cochlear injury at the molecular level, supporting the idea of diabetes as a metabolic ototoxin.

Several limitations should be considered when interpreting this study. First, the STZ model primarily represents type 1 diabetes and may not fully capture the insulin resistance and lipid dysregulation observed in type 2 diabetes. Future studies using high-fat-diet-induced insulin resistance, genetic type 2 diabetes models, or combined high-fat diet/low-dose STZ paradigms will be necessary to determine whether the identified transcriptomic signatures are conserved across diabetic phenotypes. Second, only male mice were used, which limits the assessment of potential sex-dependent differences in diabetes-associated auditory dysfunction and cochlear gene expression. Third, this study examined a single 20-week endpoint; therefore, it cannot determine the onset, progression, or temporal sequence of ABR threshold elevation and transcriptomic alterations. Longitudinal studies across multiple time points will be needed to determine whether the observed molecular changes precede, accompany, or follow auditory dysfunction.

Additional technical limitations relate to the transcriptomic analysis. Our bulk RNA-seq approach provides regional resolution, but it lacks cell-type specificity. Integration with single-cell RNA-seq or spatial transcriptomics would be valuable to further dissect cellular contributors to diabetic cochlear injury. Although sample clustering was assessed by PCA and no apparent technical batch-associated clustering was observed, the possibility of unrecognized batch effects cannot be completely excluded, particularly given the small sample size of this exploratory RNA-seq study. Future studies with larger cohorts and experimental designs incorporating explicit batch-control strategies will be important to confirm the reproducibility of the identified transcriptomic signatures. Because the present study primarily relies on ABR assessment and bulk RNA-seq analysis, the transcriptomic findings should be interpreted as exploratory and hypothesis-generating. Although the enriched pathways suggest involvement of inflammatory, immune-related, metabolic stress, and membrane-associated processes, this study does not directly demonstrate immune-cell infiltration, oxidative stress, neuronal degeneration, or histological cochlear injury. Future validation using quantitative PCR, protein-level assays, immunohistochemistry, histopathological assessment, and cell-type-resolved transcriptomic approaches will be necessary to determine the cellular origin and functional significance of the identified gene expression changes. Another important limitation is the small sample size used for RNA-seq analysis, with three biological replicates per group. Although this sample size is commonly used in exploratory transcriptomic studies of micro-dissected cochlear tissues, it limits statistical power and may increase the possibility of false-positive or false-negative differential expression findings. Therefore, the identified DEGs and enriched pathways should be interpreted as exploratory and hypothesis-generating rather than definitive mechanistic evidence. Future studies using larger independent cohorts, independent RNA-level validation such as qPCR or RNAscope, and protein- or tissue-level validation such as immunohistochemistry will be necessary to confirm the reproducibility and biological significance of these transcriptomic signatures. A further limitation of this study is that the RNA-seq results were not validated using an independent method such as qPCR, Western blotting, immunohistochemistry, or RNAscope. Because the cochlear samples were obtained by micro-dissection and the available RNA was limited, additional validation could not be performed in the present pilot study. Therefore, the identified DEGs and enriched pathways should be interpreted as exploratory transcriptomic signatures that require future confirmation at the RNA and protein levels.

This pilot study provides one of the first region-specific transcriptomic analyses of the diabetic mouse cochlea and shows that STZ-induced diabetes is associated with ABR threshold elevation and cochlear gene expression alterations. The identification of a common set of inflammatory and stress-response genes strongly supports the concept that diabetes may share transcriptomic features with other cochlear stress conditions, including ototoxic or noise-related injury models, inducing a conserved cochlear injury signature. These findings offer mechanistic insights into diabetic hearing loss and establish a foundation for the development of toxicity biomarkers and otoprotective strategies in the context of metabolic disease.

## 5. Conclusions

This pilot study shows that STZ-induced diabetes is associated with ABR threshold elevation and region-specific gene expression alterations in the mouse cochlea. These identified transcriptional changes suggest the involvement of inflammation, immune-related, metabolic stress, and membrane-associated pathways, with the lateral wall appearing to be the most transcriptionally responsive cochlear region in this model. Although these findings do not directly demonstrate cochlear structural injury or therapeutic efficacy, they provide a transcriptomic resource and a framework for future studies aimed at validating the cellular mechanisms underlying diabetes-associated auditory dysfunction and exploring potential strategies for preventing diabetes-associated hearing loss.

## Figures and Tables

**Table 1:** Shared differentially expressed genes across cochlear regions in STZ-induced diabetic mice. Fold-change ratios were converted to signed log_2_ fold changes to improve interpretability, particularly for genes with very low baseline expression in control samples. Positive values indicate higher expression in diabetic mice, whereas negative values indicate lower expression in diabetic mice. M, modiolus; L, lateral wall; O, organ of Corti; D, diabetic group; C, control group.

Upregulated Genes in Diabetic Group						
Gene Name	*p* Value—D vs. C (M)	log_2_FC D/C (M)	*p* Value—D vs. C (L)	log_2_FC D/C (L)	*p* Value—D vs. C (O)	log_2_FC D/C (O)
*Saa3*	6.66 × 10^−8^	22.97	1.79 × 10^−6^	11.20	1.05 × 10^−2^	6.58
*Irg1*	9.38 × 10^−3^	21.54	1.41 × 10^−5^	22.53	2.13 × 10^−2^	8.11
*Cd300lf*	7.17 × 10^−10^	20.80	2.89 × 10^−2^	2.76	1.66 × 10^−2^	2.72
*Gm10872*	1.02 × 10^−2^	17.98	1.87 × 10^−2^	17.97	2.73 × 10^−2^	17.67
*Il1rn*	1.51 × 10^−7^	7.20	6.96 × 10^−3^	9.00	2.76 × 10^−3^	4.26
*Slfn4*	9.06 × 10^−6^	6.84	1.17 × 10^−4^	5.11	1.69 × 10^−2^	3.64
*Il1r2*	4.84 × 10^−3^	6.14	1.09 × 10^−3^	4.87	1.17 × 10^−3^	18.24
*Cxcl13*	2.71 × 10^−2^	5.66	9.40 × 10^−3^	2.56	9.54 × 10^−4^	2.44
*Stfa2l1*	1.59 × 10^−2^	5.62	5.18 × 10^−3^	21.48	1.12 × 10^−2^	21.01
*Igj*	3.21 × 10^−2^	5.05	1.18 × 10^−2^	9.49	4.92 × 10^−12^	22.62
*Tnf*	3.09 × 10^−2^	4.39	4.57 × 10^−3^	19.54	1.60 × 10^−3^	19.75
*Clec4n*	7.67 × 10^−3^	3.79	1.94 × 10^−2^	2.28	7.50 × 10^−3^	3.82
*Apoc4*	2.66 × 10^−2^	3.74	6.85 × 10^−3^	20.21	2.03 × 10^−2^	4.10
*S100a8*	2.78 × 10^−7^	3.18	5.35 × 10^−6^	3.75	1.35 × 10^−2^	1.82
*Igk*	2.55 × 10^−3^	3.13	4.78 × 10^−3^	4.66	6.39 × 10^−4^	2.92
*Ccl9*	1.61 × 10^−3^	2.53	1.57 × 10^−3^	2.91	4.32 × 10^−4^	2.55
*Ccl6*	3.71 × 10^−4^	2.46	5.65 × 10^−4^	3.00	2.76 × 10^−4^	3.41
*Ms4a6d*	3.33 × 10^−4^	2.31	2.61 × 10^−5^	3.05	1.25 × 10^−3^	2.20
*Pirb*	3.10 × 10^−2^	1.62	1.00 × 10^−3^	2.54	1.25 × 10^−2^	1.97
*Mmp8*	4.32 × 10^−2^	1.46	6.80 × 10^−3^	4.31	2.25 × 10^−3^	4.61
*Lilrb4*	2.89 × 10^−2^	1.46	5.21 × 10^−3^	1.84	2.73 × 10^−3^	2.21
*Dynlt1b*	2.43 × 10^−4^	1.35	4.50 × 10^−3^	1.04	3.39 × 10^−2^	1.01
*Fcgr2b*	5.73 × 10^−3^	1.32	2.01 × 10^−3^	2.03	3.29 × 10^−6^	3.03
*Itgam*	1.57 × 10^−2^	1.26	2.73 × 10^−2^	1.07	8.82 × 10^−3^	1.93
*Cd14*	2.05 × 10^−2^	1.24	1.47 × 10^−3^	1.96	1.48 × 10^−2^	1.68
*Ifitm1*	2.63 × 10^−2^	1.20	7.47 × 10^−4^	1.90	1.50 × 10^−3^	1.01
*Msr1*	5.10 × 10^−3^	1.18	2.03 × 10^−2^	1.20	8.88 × 10^−3^	2.05
*Tlr13*	3.76 × 10^−2^	1.11	1.15 × 10^−2^	1.76	2.80 × 10^−3^	2.04
*C5ar1*	2.74 × 10^−2^	1.04	4.19 × 10^−2^	1.01	2.84 × 10^−3^	3.06
*Lyz2*	1.66 × 10^−2^	1.02	4.04 × 10^−2^	1.04	2.12 × 10^−2^	1.42
**Downregulated Genes in Diabetic Group**						
Gene Name	*p* value—D vs. C (M)	log_2_FC D/C (M)	*p* value—D vs. C (L)	log_2_FC D/C (L)	*p* value—D vs. C (O)	log_2_FC D/C (O)
*Hspa1b*	2.05 × 10^−2^	−1.20	2.25 × 10^−2^	−1.89	2.33 × 10^−3^	−1.93
*Lrrc73*	9.58 × 10^−3^	−1.37	4.04 × 10^−2^	−1.14	3.99 × 10^−2^	−1.41

## Data Availability

Data are contained within the article. The raw data files generated by NGS analysis in this study have been deposited in the Gene Expression Omnibus (GEO) repository with the accession number GSE303895.
